# Radiomics and Magnetic Resonance Imaging of Rectal Cancer: From Engineering to Clinical Practice

**DOI:** 10.3390/diagnostics11050756

**Published:** 2021-04-23

**Authors:** Francesca Coppola, Valentina Giannini, Michela Gabelloni, Jovana Panic, Arianna Defeudis, Silvia Lo Monaco, Arrigo Cattabriga, Maria Adriana Cocozza, Luigi Vincenzo Pastore, Michela Polici, Damiano Caruso, Andrea Laghi, Daniele Regge, Emanuele Neri, Rita Golfieri, Lorenzo Faggioni

**Affiliations:** 1 Department of Radiology, IRCCS Azienda Ospedaliero Universitaria di Bologna, 40138 Bologna, Italy; francesca_coppola@hotmail.com (F.C.); silvia.lomonaco@studio.unibo.it (S.L.M.); arrigo.cattabriga@me.com (A.C.); adrianacocozza1992@gmail.com (M.A.C.); luigiv.pastore@gmail.com (L.V.P.); rita.golfieri@unibo.it (R.G.); 2 Department of Surgical Sciences, University of Turin, 10124 Turin, Italy; valentina.giannini@ircc.it (V.G.); jovana.panic@ircc.it (J.P.); defeudis.arianna@gmail.com (A.D.); daniele.regge@ircc.it (D.R.); 3 Diagnostic and Interventional Radiology, Department of Translational Research, University of Pisa, 56126 Pisa, Italy; emanuele.neri@med.unipi.it (E.N.); lfaggioni@sirm.org (L.F.); 4 Radiology Unit—Sant’Andrea University Hospital, Department of Surgical and Medical Sciences and Translational Medicine, Sapienza University of Rome, 00189 Rome, Italy; michela.polici@uniroma1.it (M.P.); damiano.caruso@uniroma1.it (D.C.); andrea.laghi@uniroma1.it (A.L.); 5 Department of Radiology, Candiolo Cancer Institute, FPO-IRCCS, Candiolo, 10060 Turin, Italy

**Keywords:** rectal cancer, surgery, neoadjuvant chemoradiation therapy, magnetic resonance imaging, radiomics, deep learning, personalized medicine

## Abstract

While cross-sectional imaging has seen continuous progress and plays an undiscussed pivotal role in the diagnostic management and treatment planning of patients with rectal cancer, a largely unmet need remains for improved staging accuracy, assessment of treatment response and prediction of individual patient outcome. Moreover, the increasing availability of target therapies has called for developing reliable diagnostic tools for identifying potential responders and optimizing overall treatment strategy on a personalized basis. Radiomics has emerged as a promising, still fully evolving research topic, which could harness the power of modern computer technology to generate quantitative information from imaging datasets based on advanced data-driven biomathematical models, potentially providing an added value to conventional imaging for improved patient management. The present study aimed to illustrate the contribution that current radiomics methods applied to magnetic resonance imaging can offer to managing patients with rectal cancer.

## 1. Introduction

With over 1.8 million new cases reported each year, rectal cancer (RC) is the third most common cancer in men and the second in women, as well as the fourth leading cause of death globally. Its overall incidence is higher in the industrialized world, but it is also rapidly increasing in developing countries [1,2,3]. Locally advanced rectal cancer (LARC) is defined as a tumor penetrating the entire bowel wall (stages 2, T3/T4N0) and/or with involvement of regional lymph nodes (stage 3, any T N1/N2); the standard of care for patients with LARC currently includes neoadjuvant chemoradiotherapy (nCRT) followed by total mesorectal excision [4,5,6].

Magnetic resonance imaging (MRI) is the most accurate imaging modality for both RC primary staging and restaging after treatment. In particular, it plays an essential role in the local staging of the disease both before and after nCRT, in that it can provide comprehensive information regarding tumor (T) and node (N) staging, extramural venous invasion (EMVI) and circumferential resection margin (CRM), which can dictate the most appropriate clinical and therapeutic approach [7,8,9,10,11]. A meta-analysis of 35 studies revealed that preoperative MRI had high diagnostic accuracy in the assessment of preoperative T staging and of CRM, boasting a sensitivity and specificity as high as 0.97 for the assessment of muscularis propria and adjacent organ invasion. Therefore, MRI is reliable in this clinical context, although significant heterogeneity exists in the literature [12].

The main challenges and pitfalls of MRI in preoperative RC staging are related to issues, such as the differentiation of early RC (cT1-T2) from LARC (cT3) and the detection of nodal metastases, which can have relevant clinical implications related to the possibility of avoiding nCRT and treating cT1 tumors with local excision. Magnetic resonance imaging has low sensitivity in distinguishing LARC extramural tumor invasion from the desmoplastic reaction, which typically appears as spiculations with a low-intensity signal on T2w imaging and no diffusion restriction on diffusion-weighted imaging (DWI) [4,13,14]. As to N-staging, MRI has been shown to have limited performance, yielding a sensitivity and specificity of approximately 70 to 80% with pathology as the gold standard [15,16]. Detering et al. [17] have recently investigated the performance of MRI in the T and N staging of early RC, showing poor accuracy in T staging (54%), with 31% and 15% of patients being clinically overstaged and understaged, respectively. Overstaging was slightly reduced (from 54.7 to 31%) with combined MRI and endorectal ultrasonography, highlighting the overall limited performance of MRI and endorectal ultrasonography in cT1 staging. The same study also confirmed the limited performance of MRI in N staging, yielding diagnostic accuracy, sensitivity and specificity of 69%, 83% and 34%, respectively, in properly identifying pN0 patients. Furthermore, a recent study by Lord et al. showed that current MRI staging for assessing T and N status in RC patients did not adequately predict prognosis, whereas specific MRI factors, such as the presence of non-nodal tumor deposits/positive EMVI status, had greater prognostic accuracy and would be superior in determining treatment and follow-up protocols [18].

The aforementioned issues stress the need to improve the diagnostic accuracy of currently available diagnostic tests by extracting as much information as possible from imaging data to minimize errors in staging and restaging and possibly gain insights regarding optimal treatment planning and prognosis [19].

The first occurrence of the term “radiomics” in the scientific literature dates back to 2012 [20,21], and, since then, radiomics has attracted increasing attention within the scientific and medical community. Radiomics aims to translate medical images into quantitative data, defined as biomarkers, which may reveal a deeper level of detail than that, which is accessible to the unaided human eye, so as to quantify tumor phenotypes, which could aid in clinical decision-making. This has promoted the idea that medical images are like “dark matter in space” since only a small percentage of imaging data are traditionally used by radiologists for the interpretation of medical images. The bulk of the information is locked up within the images themselves unless advanced algorithms are used to unveil it and find potential correlations with biologically and/or clinically relevant factors, including early response to specific treatments, eligibility to target therapies and individualized patient outcomes [9,22,23,24]. This also holds true for RC patients, for whom radiomics can be a promising approach to improving patient care and optimizing healthcare resources. Of note, radiomics has the potential of bringing a significant contribution to tailoring treatment to the specific tumor biology of individual RC patients, possibly finding quantitative associations between radiological imaging and specific biomarkers (e.g., KRAS mutation status, microsatellite instability) from the entire tumor or any part of it, which can be exploited for target therapies [25,26,27].

Some recent studies have supported the role of radiomics in RC staging with the goal of overcoming the limitations of conventional imaging and providing an additional imaging biomarker, allowing for the correct management of RC patients [28,29].

The aim of this paper was to give an overview of the biophysical background, technical issues and potential applications of radiomics in the management of patients with RC. To this purpose, we searched PubMed (https://pubmed.ncbi.nlm.nih.gov/, accessed on 8 April 2021) for English language papers (including original research and review articles) with keywords, including “radiomics”, “rectal cancer”, “rectal MRI”, “staging”, “neoadjuvant (chemoradiation therapy)”, “surgery”, “prognosis”, “outcome”, “downstaging”, “response” and “lymph node/nodal”. For technical aspects, we also searched PubMed and IEEE Xplore (https://ieeexplore.ieee.org/Xplore/home.jsp, accessed on 8 April 2021) for English language papers with keywords, including “features standardization”, “deep learning architecture”, “machine-learning algorithms”, “feature selection”, “classifiers”, “validation techniques” and “deep learning/machine learning studies design”.

## 2. Radiomics Workflow

**Figure 1:**
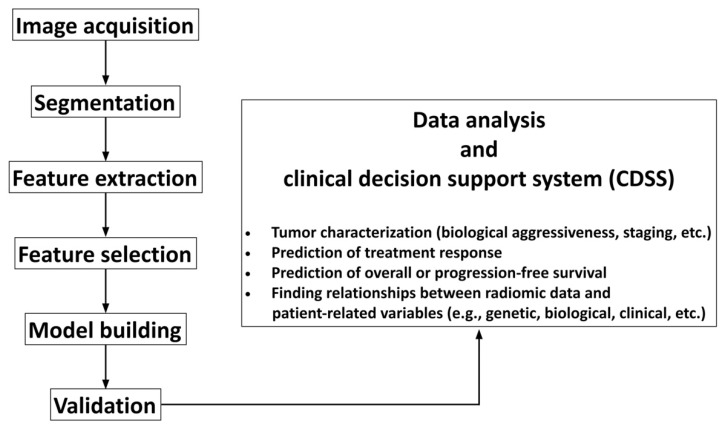
Flowchart illustrating the basic steps of radiomics workflow.

The radiomics workflow is composed of various steps, which are summarized in Figure 1 and are discussed below.

### 2.1. Image Acquisition, Segmentation and Feature Extraction

Sharing common imaging protocols across centers is of paramount importance to minimize any potential bias, affecting measurements (e.g., magnet strength, MR coil, the field of view, spatial resolution, various MR sequence parameters, etc.) [30]. However, in a clinical setting, it might be difficult to use standardized protocols on a multicenter basis, especially in the case of retrospective studies [31]. Different solutions have been proposed to reduce the influence of acquisition protocols on radiomics studies. One approach is based on leveraging radiomics variability as a feature selection tool by using test–retest analysis and eliminating radiomics features with a high variability [23,32]. However, this approach has the drawback of not evaluating the diagnostic performance of each feature. In fact, it has been demonstrated that features robust to parameter variations commonly resulted in a robust, but not necessarily high, diagnostic performance [33], whereas highly variable radiomics features could maintain a high diagnostic performance [34].

A different strategy is to apply post-reconstruction batch harmonization techniques to minimize feature variabilities across centers, such as global scaling (min–max scaling), z-standardization, the ratio with the signal intensity of healthy tissue, which is not affected by the disease, histogram-matching (where intensity histograms are transformed to match a reference intensity histogram) or the ComBat harmonization method [35]. Each of these techniques has its own strengths and weaknesses, which could affect both the values of the radiomics features and the prognostic value of radiomics-based classification methods differently [35]. Therefore, a thorough assessment of the most appropriate technique to be used is advised when developing radiomics models for multicenter studies.

**Figure 2:**
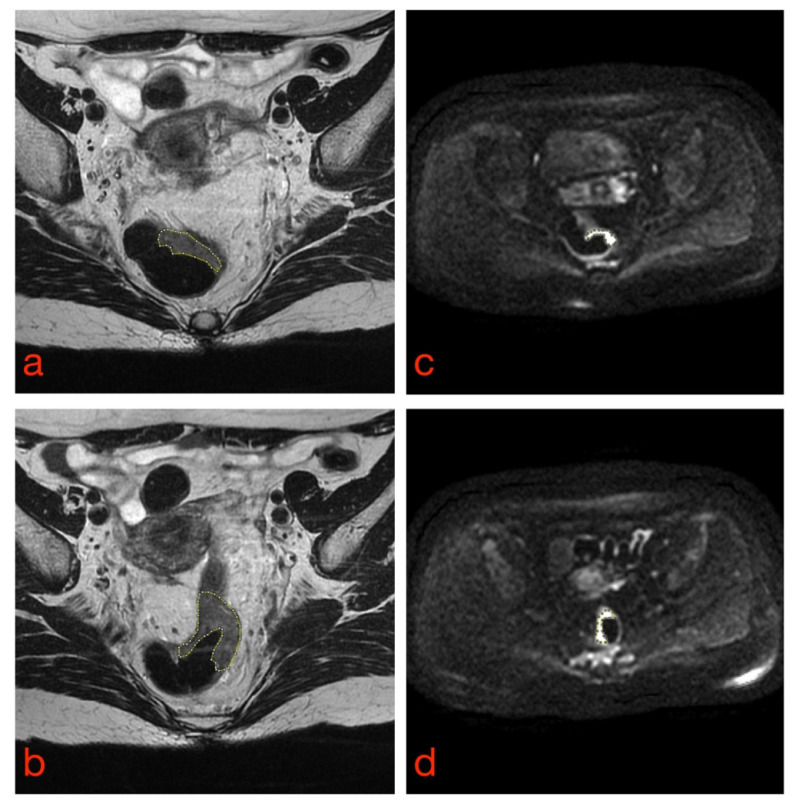
Manual tumor segmentation from T2-weighted (**a**,**b**) and DW (**c**,**d**) rectal MR images. Tumor borders are highlighted as dashed lines.

Once all image datasets have been acquired, the tissues of interest must be outlined by tracing regions of interest (ROIs) inside or around them to differentiate them from neighboring structures (e.g., tumor vs. normal tissue) (Figure 2). This task is called segmentation and can be manual, semiautomatic or fully automatic. Manual and semiautomatic methods are time-consuming tasks, hence difficult to implement in daily radiological and clinical practice; they also suffer from high intra- and interobserver variability [20]. Several studies have demonstrated that differences among segmentations can strongly affect the values of radiomics features and, consequently, the robustness of the radiomics model [36,37,38]. Therefore, when developing classifiers based on segmentation of ROIs, it is essential to determine the variability of radiomics features concerning the tumor delineation process and to implement the appropriate strategies to deal with this issue, e.g., by eliminating those features whose variability is higher than their prognostic value.

### 2.2. Automatic Segmentation via Deep Learning Algorithms

Fully automated segmentation methods have the potential of overcoming the aforementioned limitations; however, the intrinsic complexity of rectal MR images, together with differences in signal intensity among different patients and different image acquisition protocols and MR scanners, can form substantial hurdles, which have long prevented developing radiomics as a tool for supporting the diagnosis. Newer deep learning (DL) systems can outperform conventional pattern recognition algorithms in segmenting RC lesions from clinical MR images [39,40,41,42,43].

While DL (also known as “deep structured learning”) represents a subgroup of machine learning techniques, it differs from the latter in that learning is fulfilled directly from data and not from ad-hoc parameters chosen beforehand.

More specifically, when ML algorithms are developed, it is mandatory to decide which quantitative features need to be extracted and used to train the network based on a priori knowledge of the problem. Conversely, DL methods are directly fed with images and automatically determine which characteristics of the images are the most important for solving the problem. In DL methods, the training phase can be either supervised (i.e., when the data provided and used from the network to learn [training] contain information regarding the class they belong to which had previously been defined by an expert) or unsupervised (i.e., when data do not contain information regarding the class they belong to).

**Figure 3:**
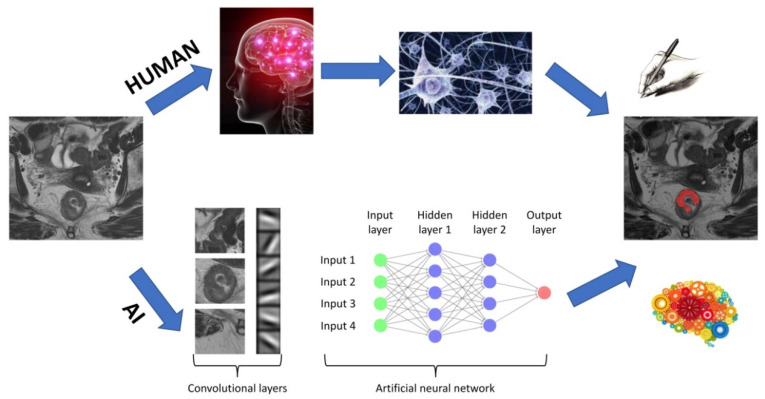
Architecture of a DL algorithm. The upper flowchart shows a human workflow, whereas the lower flowchart shows the steps needed for artificial intelligence (AI) to accomplish the same task.

The core of DL is in artificial neural networks, the main components of which are illustrated in Figure 3.

Briefly, an input image is divided into feature maps (i.e., convolutional layers) representing different characteristics of the same image. Each convolutional layer detects different levels of features from simple patterns, such as edges and gradients, to more abstract features. These feature maps are then fed into a net, which processes each of these features and activates those neurons, which are related to the recognition of a specific pattern. Multiple architectures of neural networks have been proposed, which differ in the following characteristics:

- Activation function of neurons, i.e., how different inputs can either activate or not a specific neuron, defined by variables, such as “weights” and “bias”;
- Architecture, defining the number of neurons and their interconnections;
- Learning algorithm, i.e., the implemented mathematical function, which allows neurons to learn.

Learning consists of varying the weights and bias values of each neuron within the network so that the system output matches the true output. The first artificial neural network was the Perceptron, which was introduced by Rosenblatt in 1958 [44]. Its structure is quite simple in that it is made up of a single neuron and can categorize the input into two classes. The Perceptron was followed by developing several additional neural networks, of which the best-known are convolutional neural networks (CNNs). Convolutional neural networks can learn directly from two- or three-dimensional images based on applying specific filters (i.e., convolutional layers in Figure 3), which extrapolate a series of specific characteristics useful for data classification. Starting from this type of network, the neural network U-net (which was specifically created for medical imaging and is characterized by a U-shaped structure) was implemented for the first time [45]. The biggest improvement brought by the U-net consists of being able to segment areas of clinical interest within the image (e.g., tumor tissue, blood vessels, etc.) instead of classifying an input image into two different classes (e.g., similar to a CNN).

**Figure 4:**
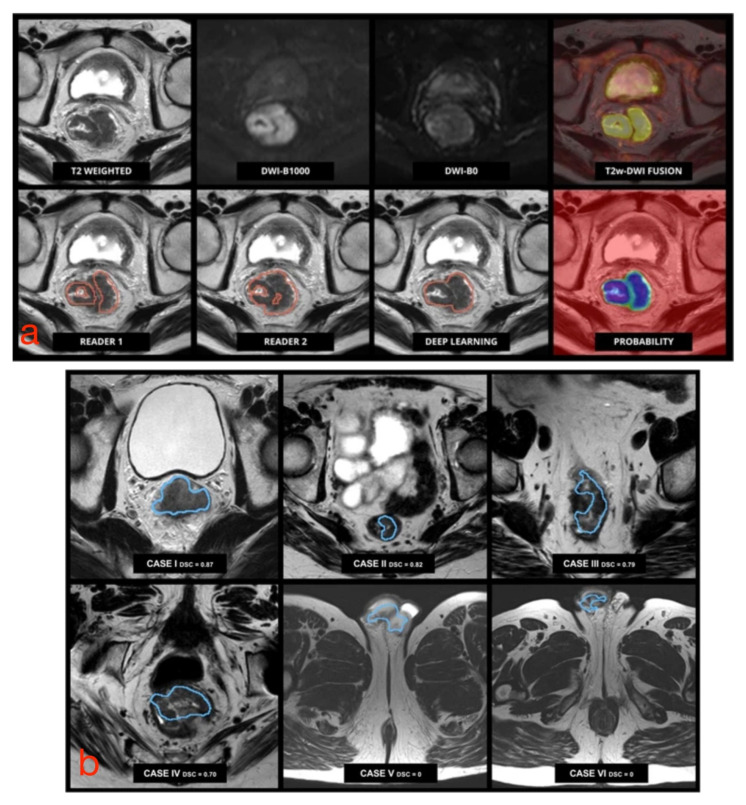
(**a**) Pretreatment multiparametric rectal MRI examination in a male patient with RC. Upper row (from left to right): axial MR images from T2-weighted images, DWI b1000, DWI b0, and fusion imaging between T2-weighted and DWI b1000 images. Lower row (from left to right): tumor segmentation performed by an experienced reader used for training, an independent reader, the algorithm output, and the corresponding probability map generated by the algorithm. (**b**) Performance of CNN-based segmentation. The algorithm correctly identified and segmented the tumor in cases I to IV (small field of view), but it failed with a larger field of view images (V and VI), where parts of the cavernous bodies of the penis were mistakenly included in the segmentation. Adapted from [46].

To date, there has been an ever-increasing number of studies showing systems based on neural networks for the segmentation of RC, given the greater interest in such technologies and their potential medical applications. The best results related to the application of CNNs have been illustrated by Trebeschi et al., who implemented a CNN-based system using multiparametric rectal MRI [46]. This system accepts T2-weighted and DW images (b-values equal to 0 and 1000 s/mm^2^) as input and is capable of classifying each voxel (3D region of the image) as neoplastic or healthy tissue (Figure 4a). The main disadvantage of this system is that its performance decreases considerably when input MR images have been acquired with a wider field of view than that of the learning dataset since portions of healthy tissue (such as testicles or subcutaneous adipose tissue) are classified as tumor tissue (Figure 4b). Huang et al. implemented a U-net, which allowed successful tumor localization on T2-weighted images [47]; however, it required a long computational time, which negated any substantial practical advantage than manual segmentation. The same authors showed the possibility of training a neural network, even using an unbalanced dataset, such as a hospital dataset in which the percentage of patients with a given disease is lower than that of healthy individuals [48]. This was possible by “weighting” the effects of pathological cases more and nonpathological cases less on the network.

Panic et al. [49] implemented an innovative system based on CNNs for automatic segmentation, which allowed overcoming the aforementioned limitations. The segmentation mask was obtained by classifying all pixel groups (ROI 3 × 3) forming the input image. The classification was carried out using three CNNs, each of them classifying the same acquired ROI from three different MRI sequences. Considering the different information provided by each individual sequence, it was possible to obtain a final classification of the region.

Current research is yielding encouraging results; however, some efforts are required to bridge the gap between research and clinical needs. First, most research algorithms are validated on internal datasets, which means that protocols and images are quite similar. Validation on larger and, especially, multicentric databases are of key importance. Furthermore, these algorithms are most often not integrated into a single graphical user interface, which can be used by an inexperienced user. Therefore, it would be a challenging but extremely important task to devise a tool, which could be run by anyone via his/her favorite reporting platform.

### 2.3. Feature Selection, Model Construction and Statistical Analysis

Once tumor segmentation has been carried out, quantitative features must be extracted, which characterize the selected tissue(s). The main features extracted are divided into first-order statistics features, which are derived from gray-level histograms, and second-order statistics features, which are computed from matrices relating each pixel of the image to those in its surroundings, e.g., using gray-level co-occurrence matrices (GLCM) or gray-level run-length matrices (GLRLM). It is essential that those steps be standardized as much as possible to avoid any bias, which may occur due to the inhomogeneity of the source data, especially in the perspective of performing multicentric studies and of future validation for clinical use [50].

A large number of variables is often extracted, resulting in the generation of features, which are highly correlated among themselves and could hence lead to overfitting (i.e., error due to over-adaptation of the prediction model to the input data), thereby requiring a prior selection of extracted features. Filter feature selection algorithms rank features according to the relationship between each feature and the output (e.g., using correlation, area under the receiver operating characteristic (ROC) curve, chi-squared test, etc.); ranking scores are then used to choose those input variables, which will be entered into the model. Conversely, wrapper feature selection methods are based on a specific machine-learning algorithm to evaluate the performance of different feature subsets. A greedy search approach is followed by evaluating all the possible combinations of features against a performance metric. For regression, this metric can be p, R-squared or adjusted R-squared values, whereas, for classification, it can be accuracy, precision, recall, f1-score, etc. Finally, the algorithm selects the combination of features yielding optimal results for the specified machine-learning algorithm. Intrinsic methods feature selection during the model building process (e.g., least absolute shrinkage and selection operator (LASSO), Elastic Net, etc.) [51].

Once the best performing features have been selected, patient clinical, biological and genetic data should ideally be incorporated into the model-building to create a complete clinical decision support system. Several classification methods can be used depending on the number of patients available and the clinical purpose (e.g., revelation and segmentation, characterization). Different classification methods have been proposed, which differ in learning approach (i.e., supervised or unsupervised) and type of correlation with the output (e.g., linear, polynomial, Gaussian, etc.). The most common classifiers are logistic regression, k-nearest neighbor, naïve Bayes classifier, support vector machines, decision tree, neural network and deep learning [52]. A recent study has concluded that there was no single best classifier across all datasets [53]; hence, the performance of different classifiers should be assessed to obtain more reliable, consistent and generalizable models.

After training the model on the data available (training set) and optimizing it on the second group of patients (testing set), its performance must be validated on a dataset possibly deriving from an external source (validation set) to evaluate whether the model created can generalize results on different settings.

Having an external validation dataset can be very difficult since the images require annotation, implying that an experienced radiologist would review each image and define the ground truth together with the oncologist and/or the pathologist. Moreover, transferring images between different centers can be problematic due to technical and privacy-related issues. A successful image exchange program can be transformational for both care providers and patients as it would allow training and validating machine learning models involving a massive scale population, thus driving better health outcomes. However, in pilot or preliminary studies, it is always mandatory to carry out validation if having an external dataset is not feasible. The latter can be accomplished using different strategies, such as k-fold, bootstrap, random subsampling or a nested approach. The biggest issue with internal validation is the potential leakage of the feature selection algorithm into the whole data, which might lead to overly optimistic results. For creating such unseen data sets, although the hold-out technique seems to be the most appropriate internal validation method, there is also a nested cross-validation technique, which is primarily used for this purpose and might give similar estimates to an independent validation [54].

## 3. Radiomics for the Personalized Management of RC Patients: Current Evidence and Perspectives

The classification models illustrated above lend themselves to providing insights into tissue structure, which can be useful to characterize tumor heterogeneity, predict patient response to therapy and overall survival, and assess the relationship between radiomic and genomic characteristics, under the assumption that imaging reflects not only the macroscopic features of tissues but also their cellular and molecular properties [55]. For the purpose of RC management, radiomics data can be obtained, which complement and/or refine the diagnostic information achieved by conventional MR protocols and image analysis.

In the future, radiomics can be expected to be an additional tool for RC patient management with the ability to support clinicians and overcoming the main challenges of conventional imaging, such as the overstaging of early RC and lack of accuracy in detecting nodal metastases. Those applications could drastically change patient management and therapeutic options, and more patients may take advantage of local excision if properly staged as T1 [28,29]. Moreover, the possibility of providing data, which go beyond the visual assessment and conventional qualitative and quantitative analysis of MR images makes radiomics a promising tool for predicting outcome before surgery and evaluating the response to nCRT (potentially allowing the selection of respondents from nonrespondents before the beginning of the completion of potentially toxic treatments), guiding towards the optimal therapeutic approach on an individualized basis [25,36,56,57]. Of note, in contrast to conventional biopsy (which samples only a selected portion of the tumor), radiomics analysis allows gathering information from the entire tumor, thus taking into account tissue heterogeneity, which may have an impact on lesion characterization and treatment planning (e.g., due to the presence of multiple tumor clones and differences in the tumor microenvironment, which may affect tumor sensitivity and resistance to nCRT or molecular target agents) [58].

### 3.1. Staging

**Figure 5:**
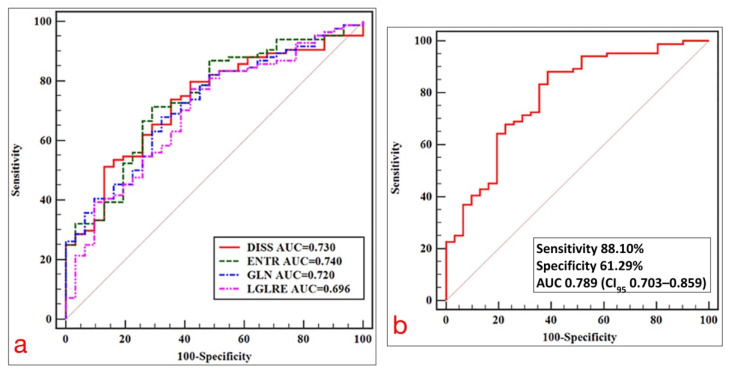
(**a**) ROC curves of statistically significant texture feature extracted from axial T2-weighted images for predicting the T-stage of RC. (**b**) ROC curve of a model for predicting T-stage based on multivariate logistic regression analysis. Adapted from [60].

In a retrospective study involving a total of 152 patients with RC, who underwent surgery alone (i.e., without any neoadjuvant therapy), Ma et al. found that radiomics analysis carried out on high-resolution T2-weighted 3-Tesla MR images using a support vector machine and a random forest algorithm yielded an area under the receiver operating characteristic curve (AUC) as high as 0.862 (sensitivity 83.3%, specificity 85.0%) and 0.746 (sensitivity 79.3%, specificity 72.2%) in predicting the degree of differentiation, and the T- and N-stages of the lesions, respectively [59]. Lu et al. reported that the texture features obtained from sagittal fat-suppression combined with transverse T2-weighted images in patients undergoing preoperative MRI may be valuable in selecting an optimal treatment strategy, allowing differentiation of T1/2 from T3/4 tumors with an AUC of 0.740 [60] (Figure 5). Similarly, Sun et al. [28] evaluated the performance of an MRI-based radiomic model for differentiating early (cT1-T2) from locally advanced (cT3-T4) RC in 119 patients, with histological diagnosis as the reference standard. The radiomic model had a superior performance as compared with MRI alone, showing a higher AUC (0.852 vs. 0.706), sensitivity (79% vs. 64.2%) and specificity (82% vs. 75.6%).

Lymph node assessment before and after treatment using conventional MRI can be challenging, spurring interest in developing advanced techniques, which could bridge this gap. Liu et al. reported that DWI-based texture analysis could predict local invasion depth (stage pT1-2 versus pT3-4) and nodal status (pN0 versus pN1-2) of RC with significant differences occurring between pN0 and pN1-2 tumors concerning the mean apparent diffusion coefficient (ADC_mean_), whereas ADC_max_ and entropy were independent predictors of positive nodal status [61]. Li et al. [29] recently built and validated a combined radiomic-clinical nomogram for predicting nodal metastases in the preoperative clinical setting of patients with colorectal cancer, achieving good accuracy, AUC, sensitivity and specificity (73.7%, 0.750, 60.2% and 84.3%, respectively).

Other studies have shown the potential of MRI texture features for providing valuable information in identifying the status of RC lymph node invasion [62,63]. A combined model derived from radiomic signatures and restaging results in patients with LARC undergoing preoperative and posttreatment MRI could predict positive lymph node status with a negative predictive value of 87.8% and 100% in posttreatment MRI T3-T4 and T1-T2 tumors, respectively [64].

### 3.2. Assessment of Treatment Response

**Figure 6:**
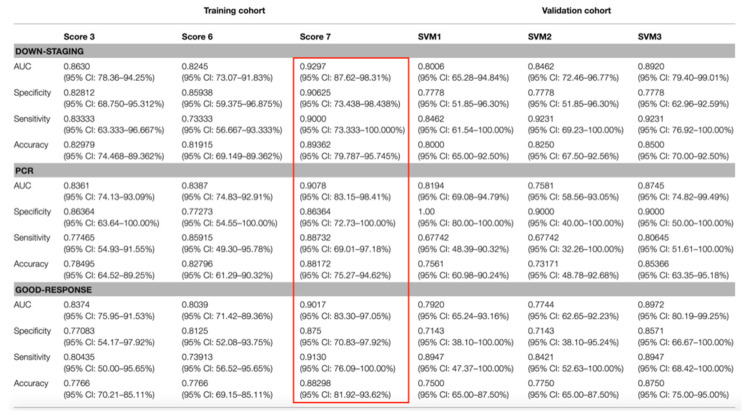
Performance of three predictive models in predicting downstaging, pathological complete response (PCR) and good response in LARC patients. The red box highlights the performance score associated with the highest AUC. Adapted from [66].

In a study by Yi et al., MRI-based radiomics was able to predict response to nCRT in patients with LARC from conventional T2-weighted MR images, allowing prediction of complete pathological response, good response (as defined by the Dowrak/Rödel system [65]) and downstaging with AUCs of 0.91, 0.90 and 0.93, respectively [66] (Figure 6).

T2-weighted images are often used because they tend to offer better diagnostic performance in RC staging [67]; however, other studies have incorporated additional sequences conveying functional information, e.g., DW images. Liu et al. built a radiomics model from T2-weighted and DW images obtained in 222 patients with LARC before and after nCRT, enabling individualized prediction of complete pathological response (AUC as high as 0.9756 in the validation cohort) and, thus, potentially aiding the identification of LARC patients, who could avoid surgery after nCRT [68]. More recently, by performing segmentation of T2-weighted and diffusion-weighted MR images (b-800 images and ADC maps), Bulens et al. created models using LASSO regression analysis, which enabled a noninvasive prediction of response to neoadjuvant treatment in patients with LARC undergoing nCRT followed by surgery, with a higher AUC between 0.83 and 0.86 for predicting complete or near-complete pathological response (ypT0–1N0), respectively [57].

An MRI-based radiomic nomogram has been developed by Wang et al., which can accurately differentiate good and poor responders in patients with LARC undergoing nCRT and achieve significant risk stratification concerning progression-free survival [69]. Zhou et al. found that pre-therapeutic, multiparametric MRI radiomic features could predict nonresponse to neoadjuvant therapy in patients with LARC, yielding an AUC of 0.822 [70]. Delta-radiomics signatures obtained in patients with LARC before and after nCRT and surgery have also been shown to be able to successfully predict treatment outcomes and serve as independent prognostic factors [71].

### 3.3. Prediction of Individual Patient Prognosis and Potential Eligibility on Target Therapies

**Figure 7:**
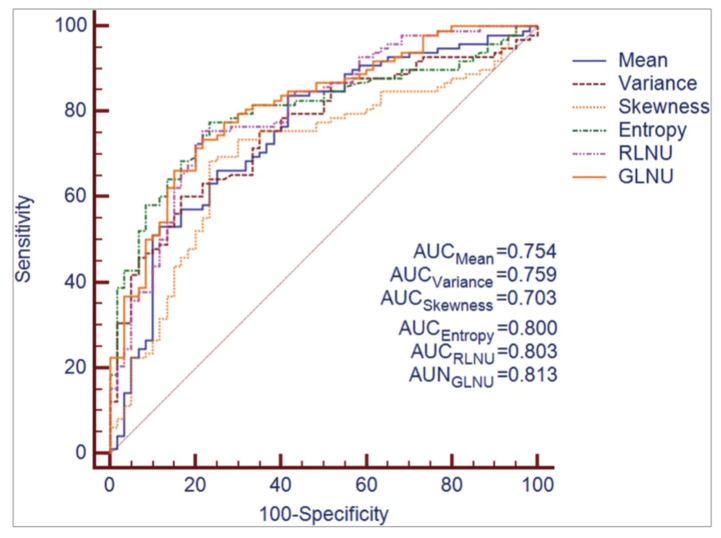
ROC curves corresponding to quantitative texture features derived from T2-weighted MR images for differentiating KRAS mutation status in rectal cancer. Adapted from [76].

Radiomics analysis may provide information, which could impact overall patient prognosis and potential eligibility for target therapies [72]. A combined model, including the radiomics and clinical features obtained from pretreatment T2-weighted MR images, was able to predict the likelihood of developing distant metastases, thus potentially aiding in tailoring treatment strategies in high-risk patients [73]. Cui et al. developed and validated a T2-weighted image-based radiomics signature for the specific prediction of KRAS mutation status in 304 patients with RC using a support vector machine algorithm providing an AUC of 0.722 in the training dataset. Importantly, a KRAS mutation was not associated with baseline clinical and histopathological features, suggesting that the proposed radiomics signature may be useful for supplementing genomic analysis to determine KRAS expression in RC patients [74]. Using T2-weighted MRI-based texture analysis, Oh et al. identified three imaging features, which could preoperatively differentiate mutant from wild-type KRAS, yielding a sensitivity, specificity and accuracy of 84%, 80%, and 81.7%, respectively [75]. In a study involving 158 patients with pathologically proven RC, who had undergone preoperative MRI, the mean values of six texture parameters (mean, variance, skewness, entropy, gray-level nonuniformity, run-length nonuniformity) were significantly higher in KRAS-mutant patients than KRAS wild-type patients having AUC values of texture features ranging from 0.703 to 0.81, and higher T stage and lower ADC values occurring in KRAS-mutant cancers [76] (Figure 7). Meng et al. developed a radiomic model from MRI datasets of 345 patients with RC taking into account multiple factors, such as lymph node metastasis, tumor differentiation grade, a fraction of Ki-67-positive tumor cells, human epidermal growth factor receptor 2 (HER-2) expression and KRAS-2 gene mutation status, yielding an AUC of 0.699 for signatures evaluating Ki-67 and of 0.697 for an integrated evaluation model incorporating radiomics signature and MRI-reported lymph node status [27].

Radiomics analysis of T2-weighted images also has the potential of identifying microsatellite instability in the preoperative setting of patients with RC [77,78]. Similar findings have also been obtained by applying radiomics methods to computed tomography (CT) [79] and dual-energy CT datasets [80].

## 4. Conclusions

Rectal cancer is a complex disease for which radiomics has the potential of providing an added value to conventional MR imaging, especially in terms of improved staging, evaluation of treatment response and prediction of patient outcome. While radiomics is an area of active research in full development, radiologists should be aware of its potential capabilities, and efforts should be made to maximize standardization of imaging protocols and data collection on a multicenter basis to validate findings on a broad scale for prospective use in clinical practice.

## References

[1] Bray F., Ferlay J., Soerjomataram I., Siegel R.L., Torre L.A., Jemal A. (2018). Global cancer statistics 2018: GLOBOCAN estimates of incidence and mortality worldwide for 36 cancers in 185 countries. CA Cancer J. Clin..

[2] Arnold M., Sierra M.S., Laversanne M., Soerjomataram I., Jemal A., Bray F. (2017). Global patterns and trends in colorectal cancer incidence and mortality. Gut.

[3] DʼSouza N., de Neree Tot Babberich M.P.M., d’Hoore A., Tiret E., Xynos E., Beets-Tan R.G.H., Nagtegaal I.D., Blomqvist L., Holm T., Glimelius B. (2019). Definition of the rectum: An international, expert-based Delphi consensus. Ann. Surg..

[4] Beets-Tan R.G.H., Lambregts D.M.J., Maas M., Bipat S., Barbaro B., Curvo-Semedo L., Fenlon H.M., Gollub M.J., Gourtsoyianni S., Halligan S. (2018). Magnetic resonance imaging for clinical management of rectal cancer: Updated recommendations from the 2016 European Society of Gastrointestinal and Abdominal Radiology (ESGAR) consensus meeting. Eur. Radiol..

[5] Gollub M.J., Arya S., Beets-Tan R.G., de Prisco G., Gonen M., Jhaveri K., Kassam Z., Kaur H., Kim D., Knezevic A. (2018). Use of magnetic resonance imaging in rectal cancer patients: Society of Abdominal Radiology (SAR) rectal cancer disease-focused panel (DFP) recommendations 2017. Abdom. Radiol..

[6] Horvat N., Carlos Tavares Rocha C., Clemente Oliveira B., Petkovska I., Gollub M.J. (2019). MRI of rectal cancer: Tumor staging, imaging techniques, and management. Radiographics.

[7] Jia X.X., Wang Y., Cheng J., Yao X., Yin M.J., Zhou J., Ye Y.J. (2018). Low- versus high-risk rectal cancer based on MRI features: Outcomes in patients treated without neoadjuvant chemoradiotherapy. AJR Am. J. Roentgenol..

[8] Taylor F.G., Quirke P., Heald R.J., Moran B., Blomqvist L., Swift I., Sebag-Montefiore D.J., Tekkis P., Brown G. (2011). Mercury study group. Preoperative high-resolution magnetic resonance imaging can identify good prognosis stage I, II, and III rectal cancer best managed by surgery alone: A prospective, multicenter, European study. Ann. Surg..

[9] Giannini V., Mazzetti S., Bertotto I., Chiarenza C., Cauda S., Delmastro E., Bracco C., Di Dia A., Leone F., Medico E. (2019). Predicting locally advanced rectal cancer response to neoadjuvant therapy with 18F-FDG PET and MRI radiomics features. Eur. J. Nucl. Med. Mol. Imaging.

[10] Ciolina M., Caruso D., De Santis D., Zerunian M., Rengo M., Alfieri N., Musio D., De Felice F., Ciardi A., Tombolini V. (2019). Dynamic contrast-enhanced magnetic resonance imaging in locally advanced rectal cancer: Role of perfusion parameters in the assessment of response to treatment. Radiol. Med..

[11] Ale Ali H., Kirsch R., Razaz S., Jhaveri A., Thipphavong S., Kennedy E.D., Jhaveri K.S. (2019). Extramural venous invasion in rectal cancer: Overview of imaging, histopathology, and clinical implications. Abdom Radiol..

[12] Zhang G., Cai Y.-Z., Xu G.-H. (2016). Diagnostic accuracy of MRI for assessment of T category and circumferential resection margin involvement in patients with rectal cancer: A meta-analysis. Dis. Colon Rectum.

[13] Nougaret S., Jhaveri K., Kassam Z., Lall C., Kim D.H. (2019). Rectal cancer MR staging: Pearls and pitfalls at baseline examination. Abdom. Radiol..

[14] Zhao R.S., Wang H., Zhou Z.Y., Zhou Q., Mulholland M.W. (2014). Restaging of locally advanced rectal cancer with magnetic resonance imaging and endoluminal ultrasound after preoperative chemoradiotherapy: A systemic review and meta-analysis. Dis. Colon Rectum.

[15] Al-Sukhni E., Milot L., Fruitman M., Beyene J., Victor J.C., Schmocker S., Brown G., McLeod R., Kennedy E. (2012). Diagnostic accuracy of MRI for assessment of T category, lymph node metastases, and circumferential resection margin involvement in patients with rectal cancer: A systematic review and meta-analysis. Ann. Surg. Oncol..

[16] Sammour T., Bedrikovetski S. (2020). Radiomics for diagnosing lateral pelvic lymph nodes in rectal cancer: Artificial intelligence enabling precision medicine?. Ann. Surg. Oncol..

[17] Detering R., van Oostendorp S.E., Meyer V.M., van Dieren S., Bos A.C.R.K., Dekker J.W.T., Reerink O., van Waesberghe J.H.T.M., Marijnen C.A.M., Moons L.M.G. (2020). Dutch ColoRectal Audit Group*. MRI cT1-2 rectal cancer staging accuracy: A population-based study. Br. J. Surg..

[18] Lord A.C., DʼSouza N., Shaw A., Rokan Z., Moran B., Abulafi M., Rasheed S., Chandramohan A., Corr A., Chau I. (2020). MRI-diagnosed tumour deposits and EMVI status have superior prognostic accuracy to current clinical TNM staging in rectal cancer. Ann. Surg..

[19] Pellino G., Gallo G., Pallante P., Capasso R., De Stefano A., Maretto I., Malapelle U., Qiu S., Nikolaou S., Barina A. (2018). Noninvasive biomarkers of colorectal cancer: Role in diagnosis and personalised treatment perspectives. Gastroenterol. Res. Pract..

[20] Lambin P., Rios-Velazquez E., Leijenaar R., Carvalho S., van Stiphout R.G.P.M., Granton P., Zegers C.M.L., Gillies R., Boellard R., Dekker A. (2012). Radiomics: Extracting more information from medical images using advanced feature analysis. Eur. J. Cancer.

[21] Kumar V., Gu Y., Basu S., Berglund A., Eschrich S.A., Schabath M.B., Forster K., Aerts H.J.W.L., Dekker A., Fenstermacher D. (2012). Radiomics: The process and the challenges. Magn. Reson. Imaging.

[22] Gillies R.J., Kinahan P.E., Hricak H. (2016). Radiomics: Images are more than pictures, they are data. Radiology.

[23] Aerts H.J.W.L., Velazquez E.R., Leijenaar R.T.H., Parmar C., Grossmann P., Carvalho S., Bussink J., Monshouwer R., Haibe-Kains B., Rietveld D. (2014). Decoding tumour phenotype by noninvasive imaging using a quantitative radiomics approach. Nat. Commun..

[24] Verma V., Simone C.B., Krishnan S., Lin S.H., Yang J., Hahn S.M. (2017). The rise of radiomics and implications for oncologic management. J. Natl. Cancer Inst..

[25] Moreira J.M., Santiago I., Santinha J., Figueiredo N., Marias K., Figueiredo M., Vanneschi L., Papanikolaou N. (2019). Challenges and promises of radiomics for rectal cancer. Curr. Colorectal Cancer Rep..

[26] Liu Z., Wang S., Dong D., Wei J., Fang C., Zhou X., Sun K., Li L., Li B., Wang M. (2019). The applications of radiomics in precision diagnosis and treatment of oncology: Opportunities and challenges. Theranostics.

[27] Meng X., Xia W., Xie P., Zhang R., Li W., Wang M., Xiong F., Liu Y., Fan X., Xie Y. (2019). Preoperative radiomic signature based on multiparametric magnetic resonance imaging for noninvasive evaluation of biological characteristics in rectal cancer. Eur. Radiol..

[28] Sun Y., Hu P., Wang J., Shen L., Xia F., Qing G., Hu W., Zhang Z., Xin C., Peng W. (2018). Radiomic features of pretreatment MRI could identify T stage in patients with rectal cancer: Preliminary findings. J. Magn. Reson. Imaging.

[29] Li M., Zhang J., Dan Y., Yao Y., Dai W., Cai G., Yang G., Tong T. (2020). A clinical-radiomics nomogram for the preoperative prediction of lymph node metastasis in colorectal cancer. J. Transl. Med..

[30] Caruso D., Zerunian M., De Santis D., Biondi T., Paolantonio P., Rengo M., Bellini D., Ferrari R., Ciolina M., Lucertini E. (2020). Magnetic resonance of rectal cancer response to therapy: An image quality comparison between 3.0 and 1.5 Tesla. Biomed Res. Int..

[31] Van Timmeren J.E., Cester D., Tanadini-Lang S., Alkadhi H., Baessler B. (2020). Radiomics in medical imaging-“how-to” guide and critical reflection. Insights Imaging.

[32] Defeudis A., De Mattia C., Rizzetto F., Calderoni F., Mazzetti S., Torresin A., Vanzulli A., Regge D., Giannini V. (2020). Standardization of CT radiomics features for multi-center analysis: Impact of software settings and parameters. Phys. Med. Biol..

[33] Lv W., Yuan Q., Wang Q., Ma J., Jiang J., Yang W., Feng Q., Chen W., Rahmim A., Lu L. (2018). Robustness versus disease differentiation when varying parameter settings in radiomics features: Application to nasopharyngeal PET/CT. Eur. Radiol..

[34] Varghese B.A., Hwang D., Cen S.Y., Levy J., Liu D., Lau C., Rivas M., Desai B., Goodenough D.J., Duddalwar V.A. (2019). Reliability of CT-based texture features: Phantom study. J. Appl. Clin. Med. Phys..

[35] Crombé A., Kind M., Fadli D., Le Loarer F., Italiano A., Buy X., Saut O. (2020). Intensity harmonization techniques influence radiomics features and radiomics-based predictions in sarcoma patients. Sci. Rep..

[36] Kim H., Park C.M., Lee M., Park S.J., Song Y.S., Lee J.H., Hwang E.J., Goo J.M. (2016). Impact of reconstruction algorithms on ct radiomic features of pulmonary tumors: Analysis of intra- and inter-reader variability and inter-reconstruction algorithm variability. PLoS ONE.

[37] Heye T., Merkle E.M., Reiner C.S., Davenport M.S., Horvath J.J., Feuerlein S., Breault S.R., Gall P., Bashir M.R., Dale B.M. (2013). Reproducibility of dynamic contrast-enhanced MR imaging. Part II. Comparison of intra- and interobserver variability with manual region of interest placement versus semiautomatic lesion segmentation and histogram analysis. Radiology.

[38] Rizzetto F., Calderoni F., De Mattia C., Defeudis A., Giannini V., Mazzetti S., Vassallo L., Ghezzi S., Sartore-Bianchi A., Marsoni S. (2020). Impact of inter-reader contouring variability on textural radiomics of colorectal liver metastases. Eur Radiol Exp..

[39] Krizhevsky A., Sutskever I., Hinton G.E. (2017). ImageNet classification with deep convolutional neural networks. Commun. ACM.

[40] Hu Z., Tang J., Wang Z., Zhang K., Zhang L., Sun Q. (2018). Deep learning for image-based cancer detection and diagnosis—A survey. Pattern Recognit..

[41] Yamashita R., Nishio M., Do R.K.G., Togashi K. (2018). Convolutional neural networks: An overview and application in radiology. Insights Imaging.

[42] Soomro M.H., Coppotelli M., Conforto S., Schmid M., Giunta G., Del Secco L., Neri E., Caruso D., Rengo M., Laghi A. (2019). Automated Segmentation of Colorectal Tumor in 3D MRI Using 3D Multiscale Densely Connected Convolutional Neural Network. J. Healthc. Eng..

[43] Coppola F., Faggioni L., Regge D., Giovagnoni A., Golfieri R., Bibbolino C., Miele V., Neri E., Grassi R. (2021). Artificial intelligence: Radiologists’ expectations and opinions gleaned from a nationwide online survey. Radiol. Med..

[44] Mullin A.A., Rosenblatt F. (1963). Principles of neurodynamics. Am. Math. Mon..

[45] Ronneberger O., Fischer P., Brox T. (2015). U-Net: Convolutional networks for biomedical image segmentation. Lecture Notes in Computer Science.

[46] Trebeschi S., van Griethuysen J.J.M., Lambregts D.M.J., Lahaye M.J., Parmar C., Bakers F.C.H., Peters N.H.G.M., Beets-Tan R.G.H., Aerts H.J.W.L. (2017). Deep learning for fully-automated localization and segmentation of rectal cancer on multiparametric MR. Sci. Rep..

[47] Huang Y.-J., Dou Q., Wang Z.-X., Liu L.-Z., Jin Y., Li C.-F., Wang L., Chen H., Xu R.-H. (2018). 3D RoI-Aware U-Net for Accurate and Efficient Colorectal Tumor Segmentation. https://arxiv.org/abs/1806.10342.

[48] Huang Y.-J., Dou Q., Wang Z.-X., Liu L.-Z., Wang L.-S., Chen H., Heng P.-A., Xu R.-H. HL-FCN: Hybrid loss guided FCN for colorectal cancer segmentation. Proceedings of the 2018 IEEE 15th International Symposium on Biomedical Imaging (ISBI).

[49] Panic J., Defeudis A., Mazzetti S., Rosati S., Giannetto G., Vassallo L., Regge D., Balestra G., Giannini V. (2020). A convolutional neural network based system for colorectal cancer segmentation on MRI images. Annu. Int. Conf. Proc. IEEE Eng. Med. Biol. Soc..

[50] Zwanenburg A., Vallières M., Abdalah M.A., Aerts H.J.W.L., Andrearczyk V., Apte A., Ashrafinia S., Bakas S., Beukinga R.J., Boellaard R. (2020). The Image Biomarker Standardization Initiative: Standardized quantitative radiomics for high-throughput image-based phenotyping. Radiology.

[51] Kuhn M., Johnson K. (2013). Applied Predictive Modeling.

[52] Koçak B., Durmaz E.Ş., Ateş E., Kılıçkesmez Ö. (2019). Radiomics with artificial intelligence: A practical guide for beginners. Diagn. Interv. Radiol..

[53] Hatt M., Parmar C., Qi J., El Naqa I. (2019). Machine (deep) learning methods for image processing and radiomics. IEEE Trans. Radiat. Plasma Med. Sci..

[54] Varma S., Simon R. (2006). Bias in error estimation when using cross-validation for model selection. BMC Bioinform..

[55] De Cecco C.N., Ganeshan B., Ciolina M., Rengo M., Meinel F.G., Musio D., De Felice F., Raffetto N., Tombolini V., Laghi A. (2015). Texture analysis as imaging biomarker of tumoral response to neoadjuvant chemoradiotherapy in rectal cancer patients studied with 3-T magnetic resonance. Investig. Radiol..

[56] Staal F.C.R., van der Reijd D.J., Taghavi M., Lambregts D.M.J., Beets-Tan R.G.H., Maas M. (2021). Radiomics for the prediction of treatment outcome and survival in patients with colorectal cancer: A systematic review. Clin. Colorectal Cancer.

[57] Bulens P., Couwenberg A., Intven M., Debucquoy A., Vandecaveye V., Van Cutsem E., D’Hoore A., Wolthuis A., Mukherjee P., Gevaert O. (2020). Predicting the tumor response to chemoradiotherapy for rectal cancer: Model development and external validation using MRI radiomics. Radiother. Oncol..

[58] Gabelloni M., Faggioni L., Neri E. (2019). Imaging biomarkers in upper gastrointestinal cancers. BJR Open.

[59] Ma X., Shen F., Jia Y., Xia Y., Li Q., Lu J. (2019). MRI-based radiomics of rectal cancer: Preoperative assessment of the pathological features. BMC Med. Imaging.

[60] Lu H.C., Wang F., Yin J.D. (2020). Texture analysis based on sagittal fat-suppression and transverse T2-weighted magnetic resonance imaging for determining local invasion of rectal cancer. Front. Oncol..

[61] Liu L., Liu Y., Xu L., Li Z., Lv H., Dong N., Li W., Yang Z., Wang Z., Jin E. (2017). Application of texture analysis based on apparent diffusion coefficient maps in discriminating different stages of rectal cancer. J. Magn. Reson. Imaging.

[62] Song L., Yin J. (2020). Application of texture analysis based on sagittal fat-suppression and oblique axial T2-weighted magnetic resonance imaging to identify lymph node invasion status of rectal cancer. Front. Oncol..

[63] Yang L., Liu D., Fang X., Wang Z., Xing Y., Ma L., Wu B. (2019). Rectal cancer: Can T2WI histogram of the primary tumor help predict the existence of lymph node metastasis?. Eur. Radiol..

[64] Zhou X., Yi Y., Liu Z., Zhou Z., Lai B., Sun K., Li L., Huang L., Feng Y., Cao W. (2020). Radiomics-based preoperative prediction of lymph node status following neoadjuvant therapy in locally advanced rectal cancer. Front. Oncol..

[65] Rödel C., Martus P., Papadoupolos T., Füzesi L., Klimpfinger M., Fietkau R., Liersch T., Hohenberger W., Raab R., Sauer R. (2005). Prognostic significance of tumor regression after preoperative chemoradiotherapy for rectal cancer. J. Clin. Oncol..

[66] Yi X., Pei Q., Zhang Y., Zhu H., Wang Z., Chen C., Li Q., Long X., Tan F., Zhou Z. (2019). MRI-based radiomics predicts tumor response to neoadjuvant chemoradiotherapy in locally advanced rectal cancer. Front. Oncol..

[67] Benson A.B., Venook A.P., Al-Hawary M.M., Cederquist L., Chen Y.-J., Ciombor K.K., Cohen S., Cooper H.S., Deming D., Engstrom P.F. (2018). Rectal cancer, version 2.2018, NCCN clinical practice guidelines in oncology. J. Natl. Compr. Canc. Netw..

[68] Liu Z., Zhang X.-Y., Shi Y.-J., Wang L., Zhu H.-T., Tang Z., Wang S., Li X.-T., Tian J., Sun Y.-S. (2017). Radiomics analysis for evaluation of pathological complete response to neoadjuvant chemoradiotherapy in locally advanced rectal cancer. Clin. Cancer Res..

[69] Wang J., Liu X., Hu B., Gao Y., Chen J., Li J. (2020). Development and validation of an MRI-based radiomic nomogram to distinguish between good and poor responders in patients with locally advanced rectal cancer undergoing neoadjuvant chemoradiotherapy. Abdom Radiol..

[70] Zhou X., Yi Y., Liu Z., Cao W., Lai B., Sun K., Li L., Zhou Z., Feng Y., Tian J. (2019). Radiomics-based pretherapeutic prediction of non-response to neoadjuvant therapy in locally advanced rectal cancer. Ann. Surg. Oncol..

[71] Jeon S.H., Song C., Chie E.K., Kim B., Kim Y.H., Chang W., Lee Y.J., Chung J.-H., Chung J.B., Lee K.-W. (2019). Delta-radiomics signature predicts treatment outcomes after preoperative chemoradiotherapy and surgery in rectal cancer. Radiat. Oncol..

[72] Caruso D., Zerunian M., Ciolina M., de Santis D., Rengo M., Soomro M.H., Giunta G., Conforto S., Schmid M., Neri E. (2018). Haralick’s texture features for the prediction of response to therapy in colorectal cancer: A preliminary study. Radiol. Med..

[73] Liu H., Zhang C., Wang L., Luo R., Li J., Zheng H., Yin Q., Zhang Z., Duan S., Li X. (2019). MRI radiomics analysis for predicting preoperative synchronous distant metastasis in patients with rectal cancer. Eur. Radiol..

[74] Cui Y., Liu H., Ren J., Du X., Xin L., Li D., Yang X., Wang D. (2020). Development and validation of a MRI-based radiomics signature for prediction of KRAS mutation in rectal cancer. Eur. Radiol..

[75] Oh J.E., Kim M.J., Lee J., Hur B.Y., Kim B., Kim D.Y., Baek J.Y., Chang H.J., Park S.C., Oh J.H. (2020). Magnetic resonance-based texture analysis differentiating KRAS mutation status in rectal cancer. Cancer Res. Treat..

[76] Xu Y., Xu Q., Ma Y., Duan J., Zhang H., Liu T., Li L., Sun H., Shi K., Xie S. (2019). Characterizing MRI features of rectal cancers with different KRAS status. BMC Cancer.

[77] Huang Z., Zhang W., He D., Cui X., Tian S., Yin H., Song B. (2020). Development and validation of a radiomics model based on T2WI images for preoperative prediction of microsatellite instability status in rectal cancer: Study Protocol Clinical Trial (SPIRIT Compliant). Medicine.

[78] Zhang W., Huang Z., Zhao J., He D., Li M., Yin H., Tian S., Zhang H., Song B. (2021). Development and validation of magnetic resonance imaging-based radiomics models for preoperative prediction of microsatellite instability in rectal cancer. Ann. Transl. Med..

[79] Fan S., Li X., Cui X., Zheng L., Ren X., Ma W., Ye Z. (2019). Computed tomography-based radiomic features could potentially predict microsatellite instability status in stage II colorectal cancer: A preliminary study. Acad. Radiol..

[80] Wu J., Zhang Q., Zhao Y., Liu Y., Chen A., Li X., Wu T., Li J., Guo Y., Liu A. (2019). Radiomics analysis of iodine-based material decomposition images with dual-energy computed tomography imaging for preoperatively predicting microsatellite instability status in colorectal cancer. Front. Oncol..

